# Structure, Performance, and Application of BiFeO_3_ Nanomaterials

**DOI:** 10.1007/s40820-020-00420-6

**Published:** 2020-03-28

**Authors:** Nan Wang, Xudong Luo, Lu Han, Zhiqiang Zhang, Renyun Zhang, Håkan Olin, Ya Yang

**Affiliations:** 1 CAS Center for Excellence in Nanoscience, Beijing Key Laboratory of Micro-nano Energy and Sensor, Beijing Institute of Nanoenergy and Nanosystems, Chinese Academy of Sciences, Beijing, 100083 People’s Republic of China; 2 School of Materials and Metallurgy, University of Science and Technology Liaoning, 185 Qianshan Zhong Road, Anshan, 114051 Liaoning People’s Republic of China; 3 School of Chemical Engineering, University of Science and Technology Liaoning, 185 Qianshan Zhong Road, Anshan, 114051 Liaoning People’s Republic of China; 4 Department of Natural Sciences, Mid Sweden University, Holmgatan 10, 85170 Sundsvall, Sweden; 5 School of Nanoscience and Technology, University of Chinese Academy of Sciences, Beijing, 100049 People’s Republic of China; 6 Center on Nanoenergy Research, School of Physical Science and Technology, Guangxi University, Nanning, 530004 Guangxi People’s Republic of China

**Keywords:** Bismuth ferrite, Multiferroic nanomaterials, Multifunctional device, Ferroelectricity, Magnetoelectric coupling

## Abstract

Multiferroic nanomaterials have attracted great interest due to simultaneous two or more properties such as ferroelectricity, ferromagnetism, and ferroelasticity, which can promise a broad application in multifunctional, low-power consumption, environmentally friendly devices. Bismuth ferrite (BiFeO_3_, BFO) exhibits both (anti)ferromagnetic and ferroelectric properties at room temperature. Thus, it has played an increasingly important role in multiferroic system. In this review, we systematically discussed the developments of BFO nanomaterials including morphology, structures, properties, and potential applications in multiferroic devices with novel functions. Even the opportunities and challenges were all analyzed and summarized. We hope this review can act as an updating and encourage more researchers to push on the development of BFO nanomaterials in the future.

## Highlights

- The development of bismuth ferrite as a multiferroic nanomaterial is summarized.
- The morphology, structures, and properties of bismuth ferrite and its potential applications in multiferroic devices with novel functions are presented and discussed.
- Some perspectives and issues needed to be solved are described.

## Graphical abstract

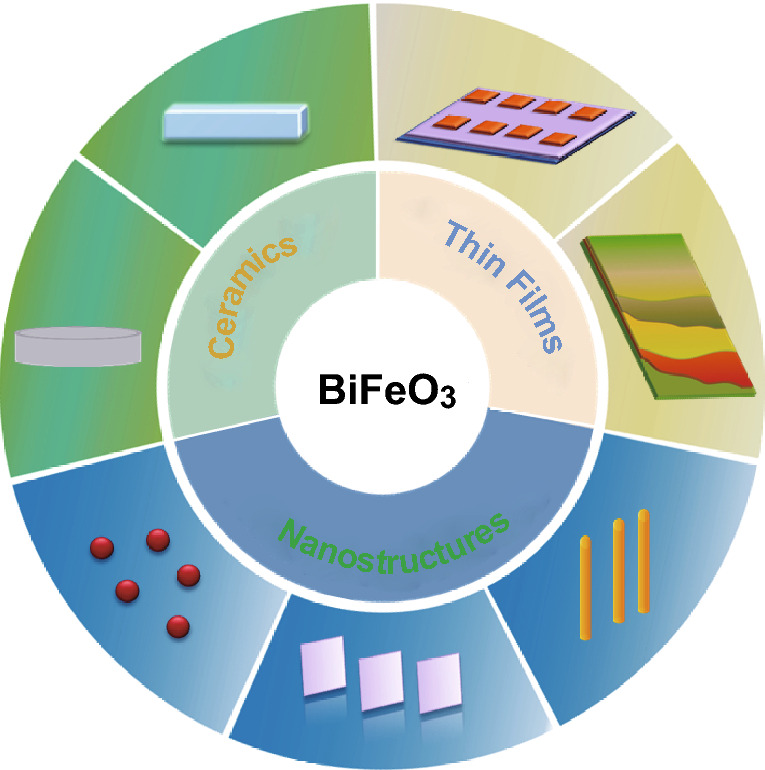

## Introduction

Multiferroic nanomaterials are materials which can possess several properties such as ferroelectricity, ferromagnetism, and ferroelasticity in single crystal [1–6]. Multiferroic nanomaterials have recently attracted great interest due to the coexistence of different order parameters in a crystalline phase. Thus, it has broad applications in multifunctional, low-power consumption, and environmentally friendly devices [4, 7, 8]. It is desirable for multiferroic nanomaterials to couple those properties at room temperature [4, 8]. However, many multiferroic nanomaterials cannot meet this need in nature. Bismuth ferrite (BiFeO_3_, BFO) has been found to have a ferroelectric phase transition Curie temperature (*T*_C_) of 1103 K and a G-type antiferromagnetic phase transition Neel temperature (*T*_N_) of 643 K, which are much higher than room temperature [9]. Due to the high *T*_C_ and *T*_N_, BFO becomes the most promising and widely known multiferroic material [7, 9].

Since it is difficult to synthesize BFO without impurity phases, it almost took decades until a significant breakthrough was made. In 2003, Wang et al. [10] firstly reported a BFO thin film with a remnant ferroelectric polarization of *P*_r_ ~ 55 µC cm^−2^, which was substantially larger than that of the reported values for single-crystal samples. Since then, BFO-based nanomaterials with remarkable versatile physical properties have been revealed in a series of forms including BFO ceramics, thin films, and nanostructures [11–15]. For example, by decreasing the leakage current high values of piezoelectric coefficient (*d*_33_) and *T*_C_ were demonstrated in BFO ceramics [13, 16]. High *P*_r_ comparable to the theoretical value was obtained in BFO thin films owning to the effect of substrate, lattice strain, or buffer layer [7, 17]. As compared with BFO ceramics and thin films, BFO nanostructures exhibit dramatic different properties. Due to the large surface area and various morphologies, BFO nanostructures exhibit significantly enhanced visible-light photocatalytic ability and magnetization. Moreover, with a low bandgap, BFO-based nanomaterials present a strong photovoltaic effect. Because of the remarkable multifunctional properties, BFO-based nanomaterials have attracted great research enthusiasm in recent years. Furthermore, the research enthusiasm will continue in the future.

This article reviews the research achievements, which have focused on the BFO-based nanomaterials in last few years. We systematically analyze the effects of structure and morphology on multiferroic properties and emphasize on enhanced functional properties for multifunctional applications. Finally, we also outlook the potentials, future development directions, and challenges of BFO-based nanomaterials.

## Forms of BFO

**Fig. 1:**
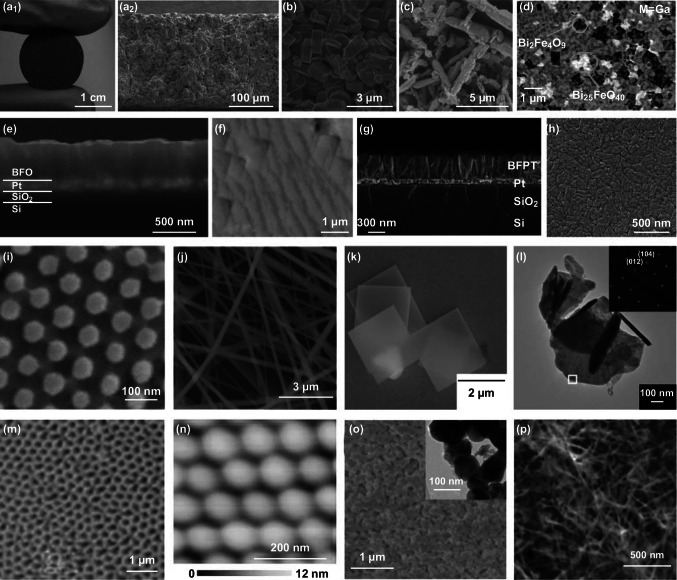
Various forms of BFO-based nanomaterials. **a**–**d** Photographs and SEM images of the BFO ceramic. Adapted with permission from Refs. [19–22]. **e**–**h** SEM images of BFO thin films. Adapted with permission from Refs. [18, 24, 25]. The morphologies of different BFO nanostructures including **i** nanodots, **j** nanofibers, **k** nanosheets, **i** nanoplate, **m** nanotubes, **n** nanoislands, **o** nanoparticles, and **p** nanowires. Adapted with permission from Refs. [31–34, 36–39]

BFO is one of the few single-phase multiferroic material, which possess of both (anti)ferromagnetic and ferroelectric properties at room temperature. Thus, it has made an important impact on multiferroic nanomaterials field. Various BFO-based nanomaterials in different forms were prepared, including BFO ceramics, thin films, and nanostructures. Here, we summarized several representative morphologies to discuss the developments of BFO-based nanomaterials (Fig. 1) [18–20].

### BFO Ceramics

After BFO ceramic was firstly synthesized in 1957, it has become one of important BFO-based nanomaterials (Fig. 1a–d) [19–22]. However, it is still challenging to synthesize a single-phase BFO ceramic because the volatility of Bi^3+^ and aggregation of oxygen vacancies can result in the occurrence of some secondary phases during the sintering process. Even more, the conversion from Fe^3+^ to Fe^2+^ and oxygen vacancies may cause a high leakage current, which makes it to be difficult for detecting a saturated ferroelectric hysteresis loop. Those factors can hinder the research of BFO ceramic. Since Bi^3+^ and Fe^3+^ are mainly responsible for the polarization and magnetization, respectively, strategies such as ion substitution (Fig. 1d) and alloying with ABO_3_ [23] compounds are induced to enhance the physical behavior. BFO ceramic with improved ferroelectric and piezoelectric properties have been obtained, and several representative morphologies are illustrated in Fig. 1a–d.

### BFO Thin Films

As compared with the difficulty to prepare a BFO ceramic with excellent ferroelectric performance, high-quality BFO thin film can be easily fabricated. Therefore, BFO thin film has attracted more and more attentions. In 2003, a BFO thin film with a large *P*_r_ of 55 µC cm^−2^ prepared on SRO/STO substrates was reported by Wang et al. [7]. Such a high *P*_r_ for BFO was firstly demonstrated, which caused a dramatic increase of research enthusiasm on BFO thin film (Fig. 1e–h) [18, 24–30]. The structure of epitaxial BFO film is closely related to the thickness of the film and the epitaxial strain on different substrate. In addition, doping other elements or forming solid solutions can also affect the structure of BFO thin films.

### BFO Nanostructures

Besides BFO ceramics and thin films, BFO nanostructures are also a research hotspot. Because of the different behaviors in photocatalyst and magnetic properties, a variety of BFO nanostructures have been reported by using different methods. Figure 1i–p displays the BFO nanostructures with different morphologies, including nanodots, nanofibers, nanosheet, nanoplate, nanotubes, nanoislands, nanoparticles, and nanowires [31–39]. The morphologies and size of BFO nanostructures largely affect the properties. It has been observed that magnetic response increases with decreasing particle sizes, and BFO nanowires are found to exhibit higher magnetization than nanorods and nanotubes [32, 40]. Moreover, the dependence of photocatalytic activity on morphology and particle size is the result of the narrow bandgap and specific surface areas.

## Structure of BFO

### Crystal Structure of BFO

**Fig. 2:**
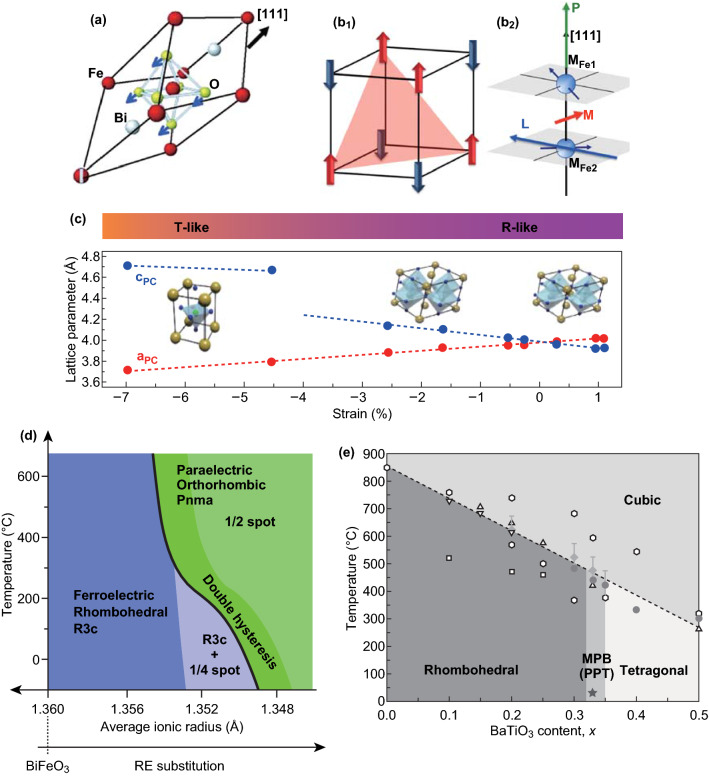
Structures of BFO and factors which influence the structure. **a** Schematic of structures of BFO unit cells. Adapted with permission from Ref. [7]. **b**_**1**_ Schematic of a G-type antiferromagnet. Spins are ferromagnetically aligned in the (111) plane, **b**_**2**_ schematically illustrating that the weak magnetization induced by canting spin cycloid structure. Adapted with permission from Ref. [95]. **c** Lattice parameters of BFO crystal structures with different strains in thin films. Adapted with permission from Ref. [71]. **d** Proposed phase diagram for rare earth-substituted BFO thin films. Adapted with permission from Ref. [46]. **e** Phase diagram of the BFO–BTO system. Adapted with permission from Ref. [13]

The crystal structure of BFO ceramic bulk is shown to possess a rhombohedral symmetry (space group R3c), with the lattice constant of *a* = 5.63 Å, and the rhombohedral angle of 89.45° at room temperature (Fig. 2a) [41]. In this structure, two perovskite cells in which the center of FeO_6_ octahedra and the angular positions are occupied by Fe^3+^ and Bi^3+^, respectively, connected along [111] direction to form the rhombohedral cell [42, 43]. The hybridization between the Bi^3+^ lone pair (6 s orbital) and O^2−^ (2*p* orbital) leads to a displacement of the Bi^3+^ from centrosymmetric positions which result in a ferroelectric polarization along [111] direction [7]. The antiferromagnetic plane (G-type) which is located perpendicular to the polarization direction comes from the Fe^3+^ [44] as displayed in Fig. 2b. The spin direction of antiferromagnetic plane is spatially modulated to form a cycloid with a period of about 62 nm [45].

Due to special places of Bi^3+^ and Fe^3+^ in the rhombohedral BFO structure, substitution Bi^3+^ and Fe^3+^ by other elements (A- and B-site substitution, respectively) may make great influence on structure and physical properties of BFO. When the ionic size and electronegativity of the dopants are similar of Bi^3+^, the dopants more tend to occupy the A-site replacing of Bi^3+^. Thus, a large number of works on the A-site substitution [46–51] pay attention on the substitution by lanthanide rare earth elements which most have a similar ionic radius, such as La, Sm, and Dy [46–48, 50–53]. Since most A-site radius is smaller than that of Bi^3+^, the smaller ions cannot fill the empty space between the FeO_6_ backbones fully and induce more buckling between the oxygen octahedra. As a result, phase structure changes are induced by substitutions. A structural transition from rhombohedral phase to an orthorhombic phase has been found in studies of rare earth-doped BFO such as Sm, Nd, Ho, and Er [48–51]. Furthermore, this structural transition was demonstrated to be a universal behavior in the rare earth-doped BFO thin films [23, 47] (Fig. 2d). By controlling the average ionic radius of the A-site cation, the structural transition with a double polarization hysteresis loop can be universally achieved [53]. Another type substitution at A-site is doped by alkaline earth ions such as Ca, Sr, and Ba which affect the BFO structure [54–56]. A structural transition from rhombohedral to triclinic was observed when 10% Ca is doped [54].

On the other hand, when the ionic size and electronegativity of the dopants are similar to that of Fe^3+^, the dopants more tend to occupy the B-site replacing of Fe^3+^ such as doping at B-site with Mn, Ti [57–64]. The valence states of Mn ions in BFO are fluctuating among +2, +3, and +4, which can make different efforts on the structure and properties of BFO. A more stable rhombohedral structure compared to BiFeO_3_ is observed when 20% Mn^4+^ is substituted, whereas mixed phases of orthorhombic and monoclinic are observed when more than 40% Fe^3+^ is substituted [57]. The addition of Mn^2+^ causes distortion in the crystals which results in a structural transition of the rhombohedral phase to the orthorhombic phase [58]. The influences of Ti doping on crystal structure, morphology, and magnetic behavior of BFO were also investigated. Rhombohedral to orthorhombic phase is observed when above 33% Fe^3+^ is substituted by Ti^4+^ [62].

Besides ion substitutions, addition of ABO_3_ to form solid solutions with BFO can dramatically modify the structure and effectively improve the properties of BFO [13, 65–67]. Among them, BiFeO_3_–BaTiO_3_ (BFO–BTO) is one typical example. In BFO–BTO, Ba acts as a large blocking ion for displacements of the Bi. Ti competes with Fe on B-site. The competitions on each site result in the formation of a continuous solid solution with different crystal structures [67]. The structure shows rhombohedral when BFO is above 67%, while below 7.5%, it shows tetragonal, while a cubic structure is observed when BFO contents between 7.5 and 67% [67]. Subsequently, a morphotropic phase boundary (MPB) was reported by Lee et al. in BFO–BTO ceramics, as shown in Fig. 2e [13]. The MPB in which rhombohedral and tetragonal phases coexist is thought to be for 0.67BFO–0.33BTO. Another solid solution of BFO formed with BiCoO_3_ was also studied systematically [28, 29, 68, 69]. Furthermore, a detailed crystal structure analysis of BiFe_1−*x*_Co_*x*_O_3_ was performed [68]. It was found that it maintains the rhombohedral *R*3*c* structure in those with *x* ≤ 0.2, while tetragonal structures are observed when *x* ≥ 0.4. Then, an MPB which is identified a monoclinic phase at approximately *x* = 0.3 is observed. It is also found that the lattice parameters of monoclinic BiFe_1−*x*_Co_*x*_O_3_ are dependent on the composition. The lattice parameters *a*/√2 and *b*/√2 decreased linearly with the increase in *x*, while the lattice parameter *c* was nearly constant. The monoclinic angle *β* also decreased with *x*.

While the bulk structure of BFO is rhombohedral, when grown on single-crystal substrates to form high-quality thin films, the structure of BFO film is strongly related to the epitaxial strain imposed by different substrates. Zeches et al. [70] revealed that epitaxial strain can be used to position BFO on a morphotropic phase and stability the structure. The various crystal structures under different strain grown on (0 0 1)-oriented substrates are summarized in Fig. 2c [71]. The BFO films present a rhombohedral-like (R-like) phase for tensile strain, while for large compressive strains, BFO films generally show a large tetragonality (T-like). Sando et al. [72] also systematically summarized the transformation progression of the BFO crystal structure from the R-like phase to T-like phase under different strain. An orthorhombic phase is stabilized when the tensile strain is beyond 2%. When a compressive strain is applied in R-like films, a transition phase is demonstrated from monoclinic *M*_*A*_ to monoclinic *M*_*C*_ by experimental characterization. When a compressive strain is applied, a mixed T–R phase from a pure R-like phase to T-like phase usually occurs. Then highly distorted tetragonal *P*_4*mm*_ phase with *c*/*a* ≈ 1.25–1.3 is observed, when the compressive strain is more than about 4.5% in the (0 0 1) orientation.

**Fig. 3:**
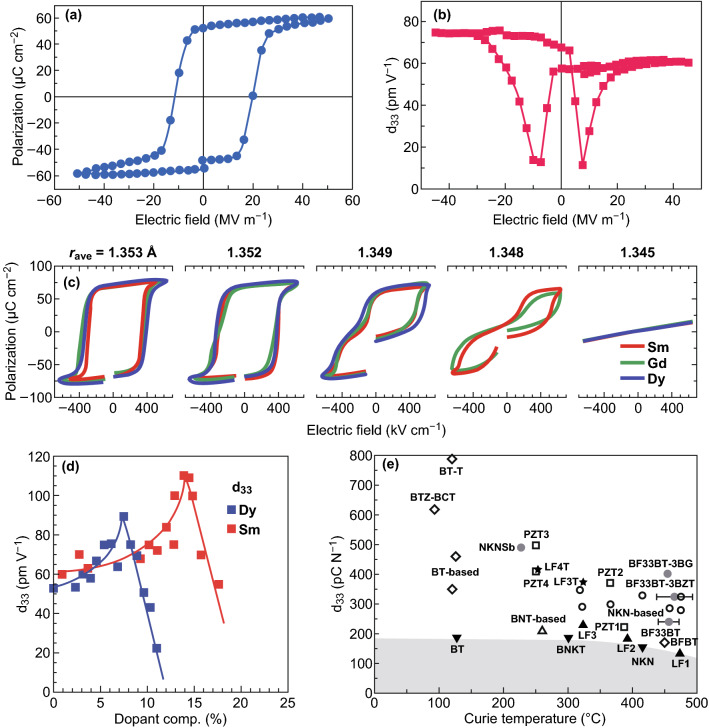
Ferroelectric and piezoelectric properties of BFO nanomaterials. **a**, **b** A ferroelectric hysteresis loop and *d*_33_ measured at an epitaxial BFO thin film. Adapted with permission from Ref. [7]. **c** Polarization hysteresis loop changes with the average A-site ionic radius changed for rare earth-substituted BFO thin films. Adapted with permission from Ref. [46]. **d** Piezoelectric coefficient *d*_33_ for rare earth-substituted BFO films. Applied Physics Letters Adapted with permission from Ref. [53]. **e** The piezoelectric properties of 0.67BF–0.33BT when a third ABO_3_ compound was added. Adapted with permission from Ref. [13]

How to choose the substrate is dependent their close lattice match to BFO and the bottom electrode. For example, a orthorhombic DyScO_3_ is chosen for the (1 1 0) crystallographic orientation which is suitable for BFO to form a (0 0 1) surface. On such substrates in a (0 0 1) orientation, BFO typically grows with a monoclinic M_A_ structure (Fig. 3c). Most of studies of BFO epitaxial thin films are prepared on STO (0 0 1) substrates, which impart a compressive strain to the film. Besides, thickness also plays an important role in the structure of BFO film. For example, ultrathin BFO films can be purely tetragonal [73, 74]. However, T-like phase may be suppressed in thicker films and tends to be R-like phase. This is because the elastic stress is relieved by the formation of the more stable R-like BFO at larger thicknesses [73]. A detailed discussion about influences of thickness and strain on the structure of BFO films can be found in a previous work [72].

## Properties of BFO

In this session, we summarize the most achievements in the properties of BFO-based nanomaterials recently, and piezoelectric, magnetic, magnetoelectric coupling, and photovoltaic properties are mainly focused on.

### Ferroelectric and Piezoelectric Properties

It can be seen from the above section that the spontaneous polarization of BFO originates from the relative shift of Bi^3 +^ from the oxygen octahedron, which causes the formation of an electric dipole moment. Due to the volatility of Bi elements and the fluctuation of valence of Fe element, a large number of oxygen vacancies and second phase are caused during the preparation of BFO, which make a sharply increased leakage current and difficulty to obtain saturated ferroelectric hysteresis loop at room temperature. It dates back to the 1970s [41] that the ferroelectric hysteresis loop of BFO ceramic bulk was first detected, with a polarization value to be 6.1 µC cm^−2^ along the [111] direction at 77 K. Then, an excitement moment was born at 2003 year. A fully saturated polarization hysteresis loop with a *P*_r_ of 55 µC cm^−2^ was obtained in a BFO thin film (Fig. 3a, b) [7]. As discussed earlier, large leakage current has hampered the development of BFO as a ferroelectric/piezoelectric material [75]. Thus, considerable efforts have been made to decrease the leakage currents for further promoting the ferroelectric and piezoelectric performance. Ion substitution and formation of solid solutions of BFO with other ABO_3_-type perovskite are most effective methods to suppress the high leakage.

In terms of ion substitution, a large number of works pay attention on the substitution by lanthanide rare earth elements. Figure 3d plots the piezoelectric coefficient *d*_33_ values as a function of the dopant composition for rare earth elements (Dy^3+^ or Sm^3+^) [53]. A dramatic increased dielectric constant up to 400 was observed at around 14% Sm composition. Concomitantly, the *d*_33_ raised to over 100 pm V^−1^. Those enhanced dielectric constant and piezoelectric coefficient are revealed to be related to the formation of a different structure: Rhombohedral phase (Sm < 14%) changes to an orthorhombic structure (Sm = 20%) [53]. In the meantime, as shown in Fig. 3c, the ferroelectric hysteresis loops of Sm-doped BFO exhibit a drastic change. A standard ferroelectric shape is shown at Sm composition < 13%, and then, it changes to a double hysteresis at Sm composition 14% and above, progressively [53]. Additional studies have shown it is a universal behavior which has also been observed in other rare earth (Dy, Gd, and La)-doped BFO [46]. It is the average radius of rare earth ionic that play the lead role in hysteresis curves, dielectric constant, and *d*_33_, which is consistent with that the phase structure changes can be caused by tuning the average A-site ionic radius, as described in Sect. 3. It further confirms the effective improvement on the ferroelectric and piezoelectric performance through modulating the phase structure by ion. Besides rare earth ionic, the ion substitution by other ion also affects the phase structure and relevant properties. For example, decreased leakage current density is found at high electric field in 3–5% Mn-doped BFO films. Well-saturated P-E loops with *P*_r_ about 100 μC cm^−2^ were observed [76]. It also found that the addition of Cr can suppress leakage current, which resulted in an increased *P*_r_ of 100 μC cm^−2^ [77].

As described in Sect. 3.1, there is an intimate link between crystal structure and the electromechanical response which means that formation of solid solutions can influence ferroelectric and piezoelectric performance of by constructing MPB. For example, a R–T MPB was confirmed in 0.67BiFeO_3_–0.33BaTiO_3_ which exhibited a high *d*_33_ value of 240 μC cm^−2^, and the *T*_c_ increase to 456 °C [13]. Then, a third ABO_3_ compound such as super-tetragonal BZT or BG was added to further improve the piezoelectric properties (Fig. 3e) [13]. The *d*_33_ values of 0.67BiFeO_3_–0.33BaTiO_3_–0.03BZT and 0.67BiFeO_3_–0.33BaTiO_3_–0.03BG were up to 324 and 402 μC cm^−2^, respectively. It is demonstrated that the addition of super-tetragonal BZT or BG can increase the structural distortions, which leads to the improvement of ferroelectric and piezoelectric properties. A Bi(Zn_2/3_Nb_1/3_)O_3_-doped BFO–BTO exhibits a low leakage (∼ 10^−7^ A cm^−2^) and effective *d*_33_^*^ (∼ 424 pm V^−1^) [78]. Doping certain element such as Ga [79] or Co [80], which can affect the microstructural parameters, such as the grain size and densification of BFO–BTO, leads to the values of *d*_33_ (157 or 167 pC/N) and *T*_C_ (467 or 488 °C). A Nb-doped BFO–BTO exhibits an effective *d*_33_^*^ which is as high as 333 pm V^−1^ [81]. In addition to BaTiO_3_, other type solid solution for BFO has also been investigated, such as PbTiO_3_ [82], SrTiO_3_ [83], or NaNbO_3_ [84], which construct different MPB. Lin et al. observed a R-M MPB in 0.75Bi_1−*x*_Nd_*x*_FeO_3_–0.25BaTiO_3_–1 mol%MnO_2_ with *x* = 0.05, giving rise to the enhanced *d*_33_ value of 121 pC/N [85]. A R-O-T MPB was also found in the solid solution BiFeO_3_–PbTiO_3_, which showed the enhanced ferroelectric and piezoelectric properties [86].

Besides, the ferroelectric and piezoelectric properties of BFO are sensitive to epitaxial strain [87]. As described previously, BFO endures a phase transition from rhombohedral to monoclinic and tetragonal phase with the increased compressive strain. Meanwhile, the polarization direction also changed. It is observed that orthorhombic BFO has its polarization in [110] directions [88], and the *M*_*A*_ phase has a polarization rotated toward [001] [87, 89]; then the polarization of T-like *M*_*C*_ phase slightly tilted away from the [001] direction in the (0 1 0) plane. There is a general trend for the polarization with strain, where the polarization along (0 0 1) plane increases with the increased compressive strain, which is consistent with that the T-like phase exhibits a much higher spontaneous polarization [72]. The theoretical *P*_r_ of the pseudo-tetragonal structure of BFO could be up to 150 μC cm^−2^ [90]. A *P*_r_ about 130 μC cm^−2^ has been reported in a super-tetragonal phase BFO film which was grown on LAO substrates [91].

In terms of piezoelectric properties, it is particularly true that there is R-like/T-like transition at around 4–5% compressive strain, which has been demonstrated to have strongly enhanced piezoelectric response [92]. It has elucidated that it is the movement of the phase boundaries between the different phases which play an important role in enhancing the piezoelectric properties [93]. The third “intermediate” polymorph acts as bridging phase in the transformation. It is not only the R-like/T-like transition can enhance the piezoelectric response. The transition between the monoclinic *M*_*c*_ and *M*_A_ phases has been demonstrated to lead to a giant *d*_33_ of 100 pm V^−1^ [94].

### Magnetic Properties

It has been demonstrated that BFO exhibited a G-type antiferromagnetic order resulted from the Fe^3+^ electron spins in the rhombohedrally distorted perovskite. Figure 2b shows the G-type antiferromagnetic lattice and the weak magnetization due to the canting of the BFO antiferromagnetic spins [95]. As shown in Fig. 2b_1_, all nearest neighbor Fe^3+^ spins point is antiparallel to one another and the spins are ferromagnetically aligned in the (111) plane. A distinguished feature of BFO magnetic order is that the G-type antiferromagnetic spin lattice cants to form a long periodicity of the spin cycloid which is 62–64 nm along the [111] direction. The incommensurate spin cycloid structure in the canted G-type antiferromagnetic arrangement permits BFO to exhibit weak magnetization below 673 K (*T*_N_) as shown in Fig. 2b_2_.

**Fig. 4:**
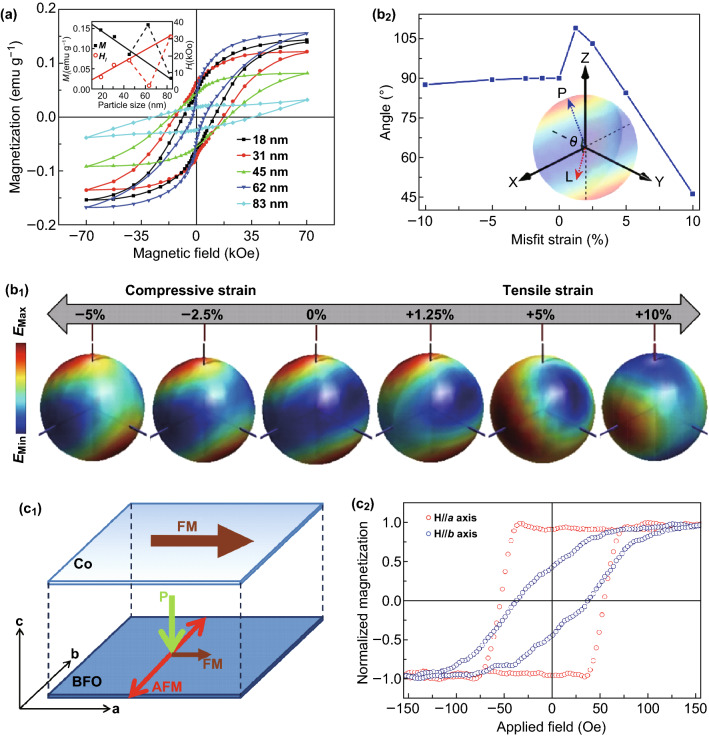
Magnetic properties of BFO nanomaterials. **a** Size effect for magnetic response of BFO nanostructure. Adapted with permission from Ref. [96]. **b**_**1**_ Three-dimensional illustration of the change of the magnetic energy landscape with different strain states. **b**_**2**_ The angle of antiferromagnetic axis and the polarization with the strain. Adapted with permission from Ref. [28]. **c** Single-domain behavior in both magnetism and ferroelectricity for BFO thin film. **c**_**1**_ Schematic diagram of the magnetization and ferroelectric polarization axes in a Co/BFO heterostructure. **c**_**2**_ Magnetic hysteresis loop of the heterostructure (red and blue circles represent the magnetic field along *a* axis and *b* axis, respectively). Adapted with permission from Ref. [29]. (Color figure online)

As being discussed earlier, the morphologies and sizes largely affect the magnetic properties so that BFO nanostructures show much better magnetic property. Figure 4a shows the size dependence of magnetic properties. The magnetic response increases dramatically below 62 nm which is the period length of the spin cycloid of BFO [96]. Studies revealed that the theoretic values of *T*_C_ and *T*_N_ depend on the particle sizes, which was also demonstrated by experimental results [97]. In terms of BFO thin films, epitaxial strain, which causes additional anisotropy, is the main factor to influence magnetic properties. Sando et al. [98] performed a systematic study of the magnetic response of strain-engineered. To eliminate the effects of thickness, the BFO films are ∼ 70 nm thick grown on various substrates. They found that the films at low compressive strain exhibited a bulk-like cycloidal spin modulation, while pseudo-collinear antiferromagnetism exists at high strain, both tensile and compressive. And the magnetic response is also changed. The low-energy magnon mode is suppressed with the increased strain. Furthermore, it is revealed that strain progressively drives the average spin angle from in-plane to out-of-plane. Recently, Chen et al. [28] have revealed that strain was used to tune the orientation of the antiferromagnetic spin, at the same time, little influence on polarization structure. As seen in Fig. 4b_1_, when the strain is free, antiferromagnetic spin axis (*L*) of the film degenerate within the plane which is perpendicular to polarization direction (along the [111]). Under compressive strain, *L* continuously rotates to point along the in-plane [$$\begin{array}{*{20}c} 1 & {\bar{1}} & 0 \\ \end{array}$$]. Under tensile strain, *L* gradually changes as the axis converges to point approximately along the [$$\begin{array}{*{20}c} 1 & 1 & {\bar{2}} \\ \end{array}$$]. When the tensile strain is further increased, *L* rotates toward the out-of-plane [110], while the polarization direction only rotates about ± 15° from the [111] within (110). Thus, antiferromagnetic axis and the polarization direction are no longer perpendicular (Fig. 4b_2_). Calculations suggest that it is Dzyaloshinskii–Moriya interaction that plays a dominant role in low strain, and the increased single-ion anisotropy at high strain results in deviation of the perpendicular relationship between antiferromagnetic axis and the polarization direction. Thus, those works predicted that strain can be used to modify the exchange bias, the magnetic orientation, and giant magnetoresistive response of spin valves which may be helpful for the development of spintronic devices.

Besides, doping elements, especially B-site replacing of Fe^3+^, can also modify the magnetic properties, such as Co [28, 69, 99], Cr [100], Ni [101], and Mn [102]. For example, a recent work has demonstrated that exchange coupling between monodomain BFO thin films and Co overlayer is intrinsic [69]. The ferromagnetic interaction between Fe^3+^ and Cr^3+^ with three electrons in *d* orbitals resulted from *d*_5_–*d*_3_ interaction. It is demonstrated that magnetic interaction between Fe^3+^ and Mn^3+^ ion along the *z*-axis is antiferromagnetic, but the other principal axes lead to ferromagnetic exchange interaction.

### Magnetoelectric Coupling

The most remarkable feature of BFO-based nanomaterials may be the magnetoelectric (ME) coupling, which can realize the coexistence and mutual coupling of ferroelectric and magnetic properties. Thus, many researchers focused on the promise how to couple the magnetic and electric order parameters [29, 103–106]. Zhao et al. [103] firstly demonstrated the electrical control of antiferromagnetic domain structure in a single-phase BFO film which indicated a strong coupling between the two types of order at room temperature. They found that the antiferromagnetic domain structure coupled strongly with the ferroelectric domain structure before and after electrical poling. The spontaneous polarization is along the [111] direction. 71°, 109°, and 180° denote three different polarization switching , and the antiferromagnetic plane is located perpendicular to the polarization direction. Meanwhile, a switching of ferroelastic domain state accompanies either the 71° or 109° polarization switching due to the change in rhombohedral axis. It is found that the coupling between antiferromagnetic domain patterns and ferroelectric domains only occurs with the 71° and 109° polarization switching, while it cannot be found with the 180° ferroelectric polarization switching. This work is a crucial first step in the exploration of approaches for researchers into investigations of the ME coupling property of BFO [35, 104, 106–108].

However, it is difficult to achieve a large magnetoelectric coupling coefficient for the magnetization from spin canting that is rather small. Then, the discovery of the magnetic anisotropy in the interface of ferromagnetic–antiferromagnetic heterostructure makes it possible to achieve a large magnetization for device applications. Additionally, thin-film heterostructures have the benefit that the magnetic anisotropy of the system can be engineered with epitaxy. Thus, researchers pay much attention on ferromagnet–multiferroic exchange coupled BFO thin-film heterostructures, especially the oxide ferromagnets/BFO heterostructures and the transition metal ferromagnet/BFO heterostructures.

La_0.7_Sr_0.3_MnO_3_ (LSMO) is a popular choice for the oxide ferromagnet/BFO heterostructures. Yu et al. [109] found different magnetic exchange effects were produced at different LSMO/BFO interface Fe^3+^ and Mn^3+^ or Mn^4+^ are ferromagnetic which competes with bulk antiferromagnetic order. Later, Wu et al. [110] demonstrated the electrical control of exchange bias using the LSMO/BFO system. By switching the ferroelectric polarization of BFO, they realized the reversible switch between two exchange-bias states. This is a great milestone for controlling magnetization with electrical control, which makes important step toward electrically controllable spintronic devices.

However, it is still a difficulty to realize the reversible electric-field control of magnetoelectric coupling at room temperature due to the correlated temperature dependence of the exchange bias in a ferromagnet/BFO heterostructure. To realize the reversible electric-field control of local magnetism at room temperature, Chu et al. [106] chose the transition metal ferromagnet (Co_0.9_Fe_0.1_) as the ferromagnetic layer to form heterojunction with BFO. They used in-plane electric field to toggle the magnetic anisotropy. They found that after the first electrical switch, the stripe-like domains change to run up from left to right. Then, an opposite electric field was applied, and the domains switched back. This series of images demonstrated that a switchable domain ferroelectric wall can be controlled by the electric field at room temperature. Most importantly, this work showed magnetoelectric switches can be in-plane 71°, which only discovered for out-of-plane 109° previously.

Besides multidomain structures, the exchange coupling between the single-domain BFO film and a ferromagnetic material was reported by Kuo et al. [29]. Figure 4c illustrates a BFO thin film that displays single-domain behavior in both magnetism and ferroelectricity [53]. As illustrated in Fig. 4c_1_, the antiferromagnetic axis is designed to be parallel to *b* axis and ferroelectric polarization parallel to *c* axis, and the ferromagnetic moment is controlled to be along *a* axis. Furthermore, on top of the BFO film, there is a Co film heterostructure. Figure 4c_2_ depicts the magnetic hysteretic loop of this Co/BFO bilayers. The clear magnetic anisotropy aligns along *a* axis, which is perpendicular to the antiferromagnetic axis of the BFO film. This clearly demonstrated that the single-domain BFO film can be used to steer the magnetic orientation. Those research results reveal that great progress has been made in development of magnetoelectric coupling which will still obtain great attention in the future.

### Optical Properties

#### Photovoltaic Effect

**Fig. 5:**
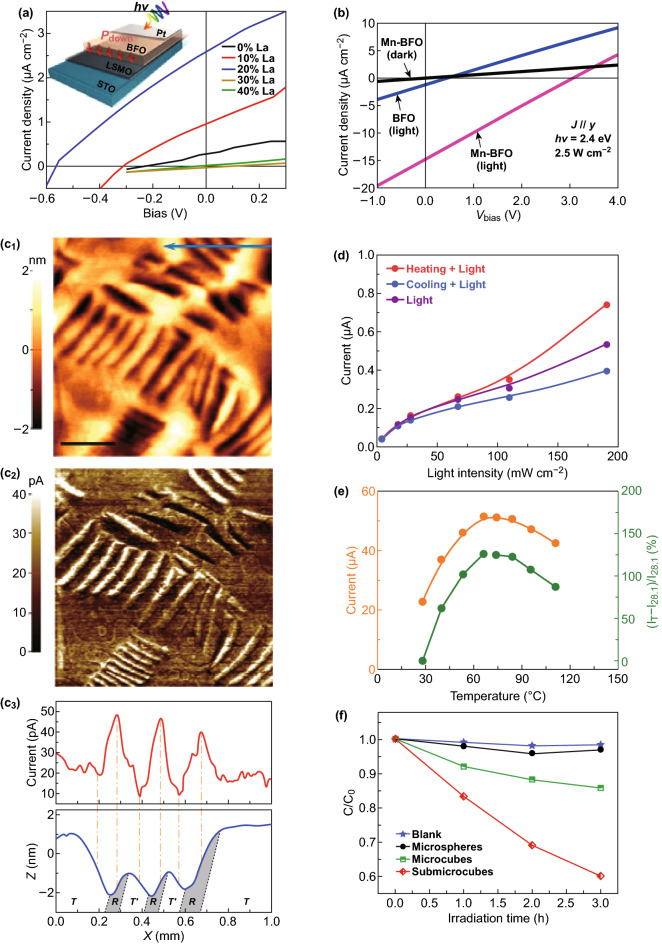
Optical properties of BFO nanomaterials. **a** Photovoltaic responses of the La-substituted BFO films. Adapted with permission from Ref. [113]. **b** Current density for both the BFO and Mn-doped BFO films. Adapted with permission from Ref. [64]. **c** Photocurrent distribution of BFO film. **c**_**1**_ Surface topography and **c**_**2**_ photocurrent distribution characterized under illumination. **c**_**3**_ Profile comparison between the photocurrent and surface morphology of the area marked by blue arrow in **c**_**1**_. Adapted with permission from Ref. [114]. **d** The photocurrent under different light intensities in heating and cooling states. Adapted with permission from Ref. [21]. **e** Output current as a function of temperature. Adapted with permission from Ref. [115]. **f** BFO nanomaterials with different morphologies show different photocatalytic activities. Adapted with permission from Ref. [40]. (Color figure online)

Since the first report of photovoltaic effect observed in BFO [111], it has been one of the most promising ferroelectric nanomaterials for photovoltaics. Completely different from the conventional photovoltaic theory, ferroelectric nanomaterials for photovoltaics have switchable photoelectric responses and above-bandgap photovoltages. In addition, ferroelectric photovoltaic belongs to the block effect which is not limited to the interface depletion layer. Researchers have reported many influences can determine the photovoltaic effect of ferroelectric nanomaterials, such as domain structure [112], polarization direction [110], and bandgap. Compared to the large bandgap of 2.8–3.5 eV of the conventional ferroelectric nanomaterials, BFO-based nanomaterials exhibit a relative narrow bandgap (< 2.7 eV) which makes it is possible for BFO-based nanomaterials to achieve more excellent photovoltaic effects. Unfortunately, the mismatch between bandgaps of ferroelectric oxides and the solar spectrum is the main obstacle to obtain high power conversion efficiency and, particularly, large photocurrent. Thus, studies on narrowing the bandgap to better match the solar spectrum is a most important branch of the research. However, this strategy always overlooked the fundamental connection between polar order and photovoltaic effect. You et al. [113] used the A-site substation in BFO to significantly enhance the ferroelectric photovoltaic effect and investigated the effect of polar order. Figure 5a illustrates the photovoltaic responses of the La-substituted BFO films under white-light illumination. 20% La-substituted film shows the highest current density. They found that with the increase of substitution ratio, the rotational instability of the polarization in the system increases, which modifies the local crystal field and band structure and drives a direct-to-indirect bandgap transition. The indirect bandgap transition can strongly inhibit the radiative recombination of the thermalized photoexcited carriers and thus enhance the ferroelectric photovoltaic characteristics. Despite being accompanied by loss of ferroelectric order, this approach offers a new strategy to enhance the photovoltaic performance in ferroelectrics.

Matsuo et al. [64] offered an approach for enhancement the photovoltaic performance without sacrificing spontaneous polarization. Using a Mn(5%)-doped BFO, they enhance not only photocurrents but also photovoltages. Figure 5b presents current density in the *y* direction under illumination. The Mn-doped BFO film shows a much higher current density than those of the BFO film. The enhanced ferroelectric photovoltaic effect is due to gap states half-filled with electrons that originate from Mn element. Under illumination with below-bandgap energy, electron–hole pairs are generated due to movement of the electrons between half-filled states which can receive electrons and the filled states that supply electrons, thus enhancing photovoltaic performance of visible-light-active photoferroelectrics.

Strain gradient can separate the light-excited electron–hole pairs and affect the photoelectric properties of photoferroelectrics as reported. Yang et al. [114] used the BFO thin films to demonstrate the role of the strain gradient in mediating photoelectric properties. We can see that the photocurrent (bright stripes in Fig. 5c_2_) is significantly enhanced at some areas of the R/T mixed-phase regions. It is also observed that the photocurrent is enhanced, while T-phase shows a negative photoconductivity by characterizing the local conduction with nanometer lateral resolution (Fig. 5c_3_). This work indicates that the strain gradient not only modulates morphotropic phase boundaries local but also controls the photoelectric performance of BFO-based nanomaterials which provide new pathways for the design of photoferroelectrics.

The role of temperature in the photocurrent BFO device was also investigated by Yong’ group [21]. Figure 5d presents the photocurrent change induced by the temperature gradient with 365-nm light illumination. An enhancement of photocurrent was observed in heating state when a stronger light was applied. Under weak light illumination, it was found that the photocurrent was enhanced in cooling state. This work demonstrated that it is possible to modulate the photovoltaic performance by controlling temperature gradients, which can provide a novel pathway to enhance the photovoltaic property of BFO-based material. Then, they further deeply investigated the relationships between photocurrent and temperature [115]. As illustrated in Fig. 5e, with the increasing temperature, the photocurrent can be first increased and then decreased. The temperature-dependent photo-sensing performance is due to the modulated energy bandgap and barrier height in the photodetector device. Those works provide new insight into the photovoltaic effect and provide a guidance for designing high-photovoltaic-performance BFO electric device.

#### Photocatalysis

Besides photovoltaics effect, bandgap of BFO nanomaterials lying in the visible-light region of the solar spectrum extends its horizons to the field of photocatalysis, especially the BFO nanostructures. Great catalytic properties of BFO nanostructures were found on different dyes such as methyl orange [116, 117], Rhodamine B [118, 119], and methylene blue [36, 120]. Many factors affect the photocatalysis behavior, such as size effect, morphologies, and the addition of a proper dopant. As illustrated in Fig. 5f, BFO nanomaterials with different morphologies show different photocatalytic activities [40], which may result from the difference in bandgap in different morphologies. Besides, particle size can significantly influence the photocatalytic property of BFO nanostructures, which may be responsible for the enhanced photocatalytic activity. Besides, BFO nanostructure with a proper dopant [121, 122] and BFO-based nanocomposites [123], which consist of different constituent nanomaterials, always exhibit the enhanced photocatalytic properties.

## Application of BFO

Due to the advantages of excellent performance, BFO materials display great potentials for designing and developing a wide variety of devices with novel functions. In this section, we will focus on the application fields of electronics, spintronics, and photonics devices.

### For Electronics

**Fig. 6:**
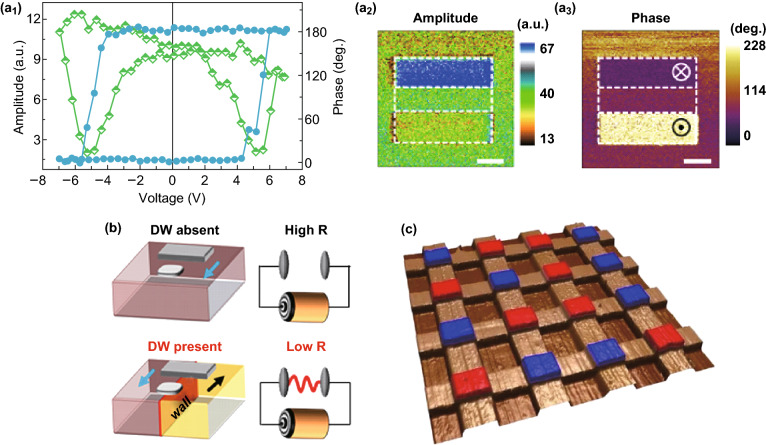
Application for electric devices. **a** Ferroelectric switching and stability in BFO films measured in argon. The applied voltages are +5 V and − 5 V voltage. Adapted with permission from Ref. [30]. **b** A prototype nonvolatile ferroelectric domain wall memory device with a scale below 100 nm. Adapted with permission from Ref. [119]. **c** Ferroelectric memory device based on photovoltaic effect of BFO. Blue: polarization up, red: polarization down. Adapted with permission from Ref. [18]. (Color figure online)

BFO-based nanomaterials have both ferroelectricity and ferromagnetism at room temperature, showing great potential for applications in high-density ferroelectric devices such as nonvolatile memories [30, 34]. However, to obtain the fast high-density, nonvolatile data storage and logic devices is dependent on a robust reversibly switchable with low operation speed, stability, and persistence [30, 35, 118]. Thus, a variety of attempts were made to tune the ferroelectric resistive switching in BFO-based nanomaterials. Using an atomically tetragonal BFO ultrathin film, Wang et al. [30] observed a stable ferroelectric polarization and its switching behavior. Figure 6a shows the switchability of the ferroelectric polarization and the out-of-plane piezoresponse force microscopy (PFM) images of 2-unit-cell (u.c)-thick BFO film. A sustained ferroelectricity performance was confirmed from the strong hysteresis behavior and butterfly-like shape when the thickness of BFO films was only 2-u.c (Fig. 6a_1_). The high-resolution PFM images further confirm the robust reversibly switchable. Furthermore, the tunneling electroresistance effect observed in ferroelectric tunnel junctions can reach ~ 370% using a 1-u.c-thick BFO film at room temperature. Such excellent performances obtained in atomically thick BFO film due to ionic displacements in oxide electrode and the surface charges will open possibilities for miniaturizing ferroelectric-based nonvolatile memories. By using a Ti-doped BFO films, Lu et al. [63] proposed an electric-optical memory prototype. This device can realize writing in electric state and reading in optical state. The films show filament-type RS effect, and the resistance state can be used to control the photovoltaic open-circuit voltage, which makes the manipulation to be repeated reliably. They also found that high-performance benefitted from the doping of Ti, which provided a feasible avenue to develop next-generation memory devices.

Another family of devices utilize ferroelectric domain walls to realize the robust reversibly switchable states with high density [34, 35, 119]. Ferroelectric domain walls are nanoscale topological defects which can separate spontaneous topological structures and be individually controlled. Thus, BFO nanostructures such as high-density and well-ordered BFO nanoislands and nanodots were proposed for use as nonvolatile memory. As illustrated in Fig. 6b, Pankaj Sharma et al. [119] demonstrated a prototype nonvolatile ferroelectric domain wall memory. This device with a scale below 100 nm can be read out nondestructively at moderate voltages of 3 V and shows a high OFF–ON ratio (≥ 10^3^). It also exhibits excellent retention and robust endurance characteristics (10^4^ and ~ 10^3^ cycles, respectively). Ma et al. [35] envisaged a cross-bar memory device using square BFO nanoislands in a self-assembled array. They found square-shaped BFO nanoislands present domains with cross-shaped charged DWs. When an electric field was applied to switch the ferroelectric polarization, the domains can be reversibly switchable between center-convergent and center-divergent states, where all polar vectors point to or away from the center. Furthermore, after the center-type domains switched to divergent states, conductance was demonstrated to enhance by three orders of magnitude. Thus, they realized reversible electric-field switching between the two stable center-type domain states. This device exhibits a polarization stability over months and a switching stability for 100 cycles. However, most of the reported switching speeds of ferroelectric-based nonvolatile memories are in microsecond level. The switching speed and the switching endurance for BFO nonvolatile memory devices still were not satisfied with the requirement of the intrinsic ferroelectric switching in universal memory which could reach the nanosecond level. Thus, BFO nonvolatile memory devices with high switching speed and excellent switching endurance still deserve more studies.

Wang et al. [18] found the photovoltaic effect of BFO was also used in the field of ferroelectric memory. It was found that both *V*_oc_ and *I*_sc_ were reversed in 10 ns which indicated a high writing speed. They also prepared a prototype 16-cell memory to demonstrate that it is feasible to construct photovoltaic effect-based ferroelectric memory (Fig. 6c).

As the growing demand for energy, an urgent need for energy storage nanomaterials with higher energy and power density rises. Recently, important progress on energy storage properties for BFO-based nanomaterials has been made. Xu et al. [124] reported a Bi_1−*x*_Nd_*x*_FeO_3_ system with theoretical predictions that energy densities and efficiencies were as high as 100–150 J cm^−3^ and 80–88%, respectively. Wang et al. [125] obtained a doped BFO–BTO ceramic energy storage system by achieving an electrically homogeneous microstructure. The discharge energy density is as high as 10.5 J cm^−3^, and efficiency is 87%.

BFO-based nanomaterials were also used for two-dimensional electron gas (2DEG). By using a BFO–TbScO_3_ (BFO/TSO) heterostructure, Zhang et al. [126] demonstrated that interfacial 2DEG was induced by ferroelectric. A strongly anisotropic conductivity dependent on polarization orientation is directly observed at BFO/TSO interface. They also found that a higher conductivity along the 109° domain stripes than in the direction perpendicular to these domain stripes. Theoretical modeling suggests the polarization-dependent interfacial conductivity is due to alternating *n*- and *p*-type conducting channels caused by domain structure. A recurring potential barrier for free carriers is built in the *p*–*n* junction, which caused an insulating interface in the perpendicular direction, whereas it also produces electron or hole along the 109° domain stripes. This work provided a new route to engineer advanced device applications, which can modulate two-dimensional anisotropic electronic transport by tuning the ferroelectric polarization.

Besides, Li et al. [127] investigated the elastic dynamics of rhombohedral–tetragonal phase transition of BFO by a tip. They found that the piezoresponse enhanced two- to threefold near this transition, concomitantly, the values of Young’s modulus decreased by over 30%. This giant electrically tunable elastic stiffness and corresponding electromechanical properties broaden potential applications of BFO toward frequency-agile electroacoustic devices.

### For Spintronic Devices

**Fig. 7:**
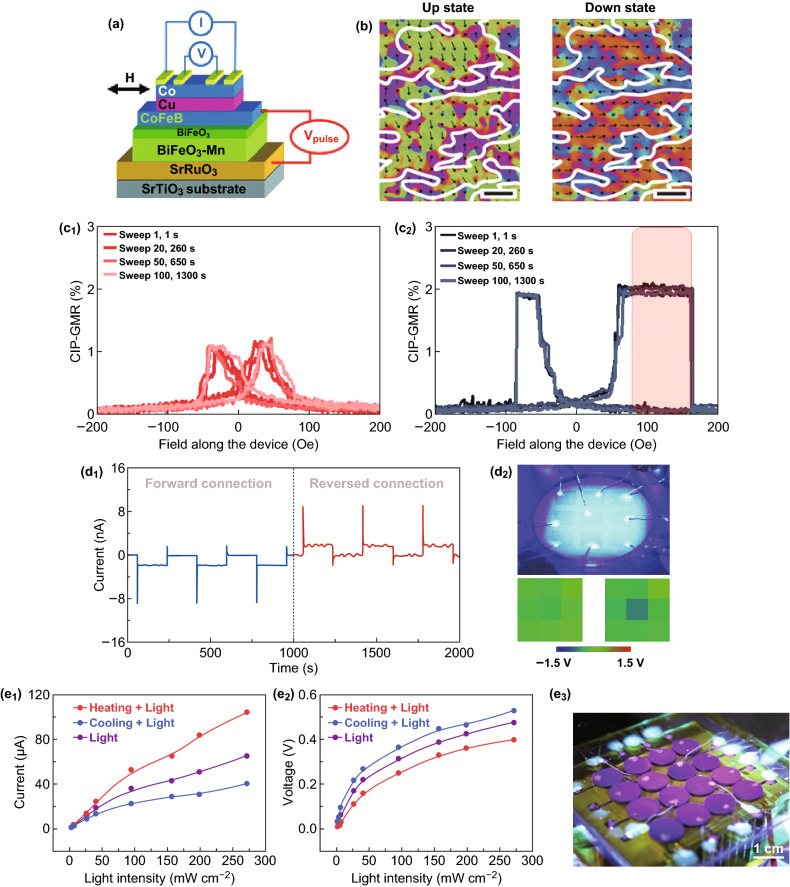
Application for spintronic and optic devices. **a** A giant magnetoresistive device based on BFO. Adapted with permission from Ref. [128]. **b** Exchange coupling between antiferromagnetic order and the ferromagnetic overlayer for BFO-based spintronic devices. Adapted with permission from Ref. [69]. **c** Voltage-controlled unidirectional anisotropy for CoFe/BFO heterostructure. A electrically switchable exchange bias is observed at the interface between the BFO and giant magnetoresistance. Adapted with permission from Ref. [108]. **d**, **e** Applications in photodetector. **d**_**1**_ Output current for ITO/BFO/Ag device. **d**_**2**_ The ITO/BFO/Ag photodetector array. Adapted with permission from Ref. [19]. **e** Photovoltaic performance of the BFO photodetector modulated by applying different temperatures. Adapted with permission from Ref. [20]

The property of magnetoelectric coupling under the room temperature results in that it is possible to control the magnetic behaviors by an electric field for BFO-based nanomaterials. As a result, BFO-based material becomes a candidate for applications in spintronic devices [107, 128]. In 2012, Allibe et al. [128] proposed a giant magnetoresistive device based on BFO by spin valve exchange to investigate the prospect of BFO-based nanomaterials in electrically written spintronic devices (Fig. 7a). The device structure was STO/SRO/BFO-Mn/BFO/CoFeB/Cu/Co, where a 1% giant magnetoresistance signal was tested. Moreover, the exchange bias was regulated by the applied voltages. However, poor stability of the BFO/CoFeB interfaces seriously hindered the increase of giant magnetoresistance signal. Later, Zhang et al. explored the thermal stability of the BFO/CoFeB interface [129] and revealed the physical mechanism. It demonstrated that the difference of oxygen vacancies induced by BFO polarization direction was the key that influenced the thermal stability. Furthermore, an effective solution was proposed to further increase the giant magnetoresistance value to 4.2%.

Sando et al. [98] studied the effect of the strain on the giant magnetoresistive response. They found that both tensile and compressive strains can induce a pseudo-collinear antiferromagnetism at high strain, which demonstrated the possibility to modify magnetic response by strain. Then, Saenrang et al. [69] investigated the exchange coupling between antiferromagnetic order and the ferromagnetic overlayer to explore BFO-based spintronic devices. They observed an intrinsic exchange coupling between BFO antiferromagnetic order and Co magnetization. As presented in Fig. 7b, in the down state, the Co uniaxial anisotropy axis is along the substrate step edges, while in the up state, it rotates nearly 90°. Furthermore, this robust and reliable switching can be reproducible for hundreds of cycles. Recently, Manipatruni et al. [108] have first demonstrated that unidirectional anisotropy was controlled by voltage for CoFe/BFO heterostructure. A robust, electrically switchable exchange bias is observed at the interface between the BFO and giant magnetoresistance. As illustrated in Fig. 7c, the exchange bias becomes stronger as the lateral dimensions are decreased at room temperature which demonstrates that the reduction in lateral dimensions of the giant magnetoresistance stack is critical to obtain exchange bias. Furthermore, they also found that the exchange bias can be reversibly modulated by a bipolar electric field. These works provide an insight into the exploiting pathway for reducing the energy per transition of magnetoelectric devices utilizing BFO.

### For Optics

Driven by growing demand for clean and renewable energy, ferroelectric photovoltaic nanomaterials, which can convert solar energy into electricity, have attracted more and more attention. As compared with the traditional ferroelectric nanomaterials, BFO-based nanomaterials present unique performances such as the narrow bandgap, better carrier transport, and light absorption characteristics. Thus, it is of significance to develop ferroelectric photovoltaic devices based on BFO nanomaterials.

Yang et al. have made outstanding works in the photovoltaic application fields based on BFO nanomaterials [44, 46, 48]. As illustrated in Fig. 7d, by using the photovoltaic–pyroelectric coupled effect, an ITO/BFO/Ag self-powered photodetector was designed to investigate potential applications of the BFO photodetector [96]. A photocurrent of about 10 nA was obtained by this photodetector. The pyroelectric effect was able to adjust the Schottky barrier height, which leads to the enhanced photocurrent. The current signal of the sharp peak in I–t curves was associated with the coupling between photovoltaic and pyroelectric effect. The steady current terrace was caused only by the photovoltaic effect. Since the pyroelectric current has been gradually exhausted, there was only the photovoltaic current when the light-induced temperature was shown to be stable. Then, a self-powered photodetector array was designed to sense 450-nm light in real time (Fig. 7d_2_). The same green color can be observed in almost all the channels when there is no light illumination. When the 450-nm light was illuminated only on pixel 5, an obvious different color can be observed in channel 5. It demonstrated that the photodetector can be used to detect 450-nm light in real time. Moreover, they also found that temperature played an important role in improvement of photovoltaic performance. As depicted in Fig. 7e, a 16-unit-based self-powered photodetector array has been developed to investigate the role of temperature on the photovoltaic effect of BFO [20]. By detecting output current and voltage signals in two states: heating and cooling, it is demonstrated that the photovoltaic performance of the BFO photodetector can be modulated by applying different temperatures. The output current can be enhanced in heating states, while the enhanced voltage response is observed in cooling states. The enhanced photocurrent is related to temperature and thermo-optical effects in photovoltaic processes. These works provide pathways to improve the photovoltaic effect in ferroelectric nanomaterials, especially for potential applications in photodetector systems.

It was also found that there was an anomalous photovoltaic effect in BFO in nanoscale. Marin Alexe et al. [130] used atomic force microscopy to investigate the photovoltaic effect in BFO single crystals. They found that the photovoltaic effect can be enhanced by seven orders of magnitude. This nanoscale enhancement is described as a tip enhancement. The top electrode with a particularly geometry causes a point-contact geometry with nanoscale dimensions which can efficiently collect the photoexcited carriers. This work might provide an insight into the physical mechanisms of photovoltaic effects in BFO-based nanomaterials.

As described in previous part, strain gradient not only modulates morphotropic phase boundaries local but also controls the optical response of BFO. Thus, Sando et al. [71] used epitaxial strain engineering to tune the optical response of BFO thin films and found that the optical index changed greatly with strain. This means a large effective elasto-optic coefficient in this system. They also observed a shift of the optical bandgap driven by strain known as piezochromism in other nanomaterials. Because this electric-controlled piezochromism can be reversible and remanent, this finding broadens the potential applications of BFO toward photonics and acousto-optic devices.

Besides, Liou et al. [131] demonstrated that it is possible to precisely control the ferroic orders by all-optical illumination at ambient temperature. As described early, the large energy mismatch between the order parameter coupling strengths and solar spectrum makes it challengeable to manipulate the ferroic orders precisely by optical methods. It is found that mixed-phase BFO composed of a tetragonal-like phase and a rhombohedral-like phase shows relatively low barrier for phase transition, which gives light-induced tuning of ferroelectricity, magnetism, and ferroelasticity opportunities. Thus, they utilized an epitaxial mixed-phase BiFeO_3_ thin film to perform the light-induced tuning of multiple ferroic orders at ambient temperature. Results indicated that light illumination can tune the domain transformation and distribution of the R–T phase. The piezoelectric property can be reversibly modulated by means of light which demonstrates that precise sequential laser writing and erasure of different domain patterns can be performed. Besides, they found that the light-induced thermal and flexoelectric effects also play important roles in manipulation of the corresponding ferroic orders. Those investigations not only shed light on the optical control of multiple functionalities but also provide theoretical and experimental basis for the future application of BFO-based material in optoelectronics applications.

## Summary and Outlook

In summary, BFO is an ideal multiferroic nanomaterial with various unique properties, for instance, huge remnant polarization, the magnetoelectric coupling at room temperature, and relatively narrow bandgap. Therefore, it serves as a versatile platform to explore new possibilities for techniques with novel functions. This article reviews the great achievements, which has been reported to investigate the structures, properties, and applications in BFO-based nanomaterials. Various strategies were proposed to modify the ferroelectric and magnetic performances. Ion substitution, selection of proper substrates, and constructing morphotropic phase boundary have commonly been used to improve the ferroelectric and magnetic performances. Size effect-induced modification of behaviors in BFO nanostructures also achieves great interests. Attempts for excellent performances have been driven to further apply in fields such as nonvolatile memories, piezoelectric sensors, and photodetector.

However, before such new devices are achieved, numerous challenges remain. Firstly, it is important to further improve the ferroelectric and magnetoelectric performances. The BFO exhibits high polarizations. Could a higher ferroelectric polarization be obtained? Could a high piezoelectric activity which can match piezoelectric ceramic transducer be attained? More significantly, can BFO devices exhibit a large magnetoelectric response at room temperature? Secondly, it involves the dynamics parameters. For example, could the responding dynamics be stabilized and could the switching speed satisfy for the devices which are designed on the basis of ferroelectric or ferromagnetic switching? Most of the reported switching speed and switching endurance still cannot be satisfied for universal memories. Therefore, it has been a long way to go by applying memory for BFO-based nanomaterials. Thirdly, the physical mechanisms of photovoltaic effect and photocatalytic behavior in BFO nanomaterials are still elusive. The scopes of BFO photocatalyst and photovoltaic devices may be further extended only if the physical mechanisms are expounded. Different theories have been proposed; however, a number of controversies still remain and further studies are deserved. Fourthly, only limited nanomaterials systems were demonstrated to show the coupling property. More nanomaterials systems still need to be developed to explore the possibilities of designing new devices. By this review, we hope to provide an updated overview on the understanding of the existing challenges and opportunities, which can further encourage more researchers to push on the development of BFO nanomaterials in the future.
